# Unraveling Structural Alerts in Marketed Drugs for Improving Adverse Outcome Pathway Framework of Drug-Induced QT Prolongation

**DOI:** 10.3390/ijms24076771

**Published:** 2023-04-05

**Authors:** Wulin Long, Shihai Li, Yujie He, Jinzhu Lin, Menglong Li, Zhining Wen

**Affiliations:** 1 College of Chemistry, Sichuan University, Chengdu 610064, China; 2 Medical Big Data Center, Sichuan University, Chengdu 610064, China

**Keywords:** drug-induced prolongation of QT interval, drug safety evaluation, structure–activity relationship, machine learning, adverse outcome pathway

## Abstract

In pharmaceutical treatment, many non-cardiac drugs carry the risk of prolonging the QT interval, which can lead to fatal cardiac complications such as torsades de points (TdP). Although the unexpected blockade of ion channels has been widely considered to be one of the main reasons for affecting the repolarization phase of the cardiac action potential and leading to QT interval prolongation, the lack of knowledge regarding chemical structures in drugs that may induce the prolongation of the QT interval remains a barrier to further understanding the underlying mechanism and developing an effective prediction strategy. In this study, we thoroughly investigated the differences in chemical structures between QT-prolonging drugs and drugs with no drug-induced QT prolongation (DIQT) concerns, based on the Drug-Induced QT Prolongation Atlas (DIQTA) dataset. Three categories of structural alerts (SAs), namely amines, ethers, and aromatic compounds, appeared in large quantities in QT-prolonging drugs, but rarely in drugs with no DIQT concerns, indicating a close association between SAs and the risk of DIQT. Moreover, using the molecular descriptors associated with these three categories of SAs as features, the structure–activity relationship (SAR) model for predicting the high risk of inducing QT interval prolongation of marketed drugs achieved recall rates of 72.5% and 80.0% for the DIQTA dataset and the FDA Adverse Event Reporting System (FAERS) dataset, respectively. Our findings may promote a better understanding of the mechanism of DIQT and facilitate research on cardiac adverse drug reactions in drug development.

## 1. Introduction

Cardiotoxicity is one of the major concerns in clinical pharmaceutical treatments [1,2,3]. Apart from cardiac drugs, a number of non-cardiac drugs may prolong the QT interval, which increases the likelihood of severe cardiac complications such as torsades de points (TdP) [4]. Understanding the mechanisms underlying the drug-induced prolongation of the QT interval is crucial, not only for preventing cardiovascular adverse events in clinical practice but also for developing new drugs in the pharmaceutical industry [5,6,7,8,9,10].

Monitoring the increase in electrocardiogram (ECG) data, along with other patient information such as gender, disease states, and concomitant use of other QT-prolonging drugs, is a common approach in clinical settings to assess the potential risk that patients will acquire a prolonged QT interval and develop TdP [2,11]. However, this approach is often hindered by individual differences [12,13]. In preclinical settings, the well-known mechanism of drug-induced QT prolongation (DIQT) is the inhibition activity of the repolarizing potassium current *I_Kr_* that is generated by the expression of the human ether-a-gogo-related gene (*hERG*) [14,15,16,17,18]. The drug-mediated blockade of the *hGRG* gene channel will cause the inhibition of rapid delayed rectifying potassium current, resulting in a prolonged QT interval and, in severe cases, developing into TdP. Based on this mechanism, the comprehensive in vitro Proarrhythmia assay (CiPA) initiative recommends screening chemical agents that may potentially prolong the QT interval by examining their effect on individual ion currents in vitro and predicting the drug effects on repolarization and proarrhythmia risk by using in silico methods [19]. Over the past decade, various machine learning methods, such as support vector machines, random forests, and Bayesian networks, have been applied to evaluate the risk of inducing cardiotoxicity of compounds by predicting the blocking effect of compounds on *hERG* gene channels [20,21,22,23,24,25,26,27,28]. Additionally, deep learning algorithms have been proposed to evaluate the risk of cardiotoxicity caused by *hERG* gene blockers and to assess the cardiotoxicity of FDA-approved small molecular drugs in the Drugbank [29,30]. Despite the relatively high predictive performance achieved by these studies, few of them provide deep insight into the chemical fragments, also known as structural alerts (SAs), in drugs that may contribute to cardiotoxicity. It is still a challenge to further study the underlying mechanism and develop an effective strategy for drug safety evaluation.

Therefore, in this study, we aim to identify the SAs in marketed drugs that prolong the QT interval based on data from the Drug-Induced QT Prolongation Atlas (DIQTA), which we developed in our previous study [31]. In DIQTA, the drugs were well stratified into different risk levels based on their potential to cause QT interval prolongation, as determined by FDA-approved drug labeling information. We thoroughly investigated the differences in chemical structures between QT-prolonging drugs and drugs with no DIQT concerns, and identified a set of SAs closely related to the high risk of prolongation of the QT interval. The structure–activity relationship (SAR) model also performed well in identifying marketed drugs with a high risk of DIQT when using the SAs as features. Our findings will promote the development of adverse outcome pathway frameworks for the drug-induced prolongation of the QT interval and facilitate research on cardiac adverse drug reactions in drug development.

## 2. Results

### 2.1. Structural Differences between QT and Non-QT-Prolonging Drugs

**Figure 1:**
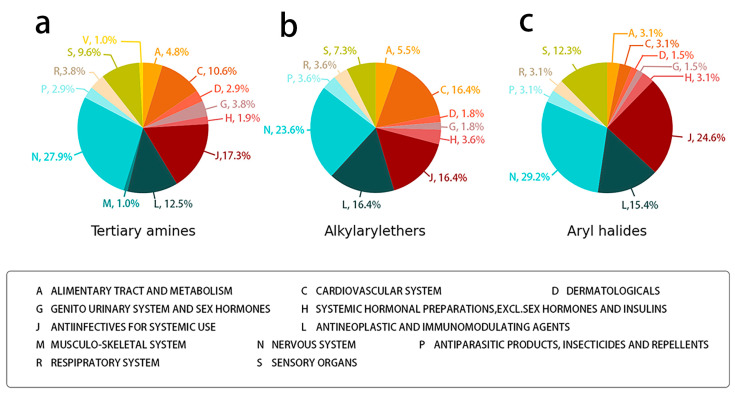
(**a**) Distribution of drugs containing tertiary amines among different therapeutic categories of QT-prolonging drugs; (**b**) distribution of drugs containing alkylarylethers among different therapeutic categories of QT-prolonging drugs; (**c**) distribution of drugs containing aryl halides among different therapeutic categories of QT-prolonging drugs.

We identified 24 chemical fragments (SAs) with large differences in chemical structures between QT-prolonging drugs and no-DIQT-concern drugs, as shown in Table 1. These 24 SAs can be broadly categorized into three groups: amines, ethers, and aromatic compounds. Amines, in particular, were found to be more prevalent in QT-prolonging drugs, with a proportion at least 30% higher than that of the no-DIQT-concern drugs. Among the amines, tertiary amines (ID: 1) had the highest difference in proportion between the two categories, with a proportion of 61.1% in the QT-prolonging drugs and only 12.6% in the no-DIQT-concern drugs. For tertiary aliphatic amines (ID: 3), they appeared in over 50% of QT-prolonging drugs, but in less than 10% of no-DIQT-concern drugs. These results suggest that amines, particularly tertiary amines, may be closely associated with drug-induced prolongation of the QT interval. Similarly, for aromatic compounds, Ethers (ID: 9) and Alkylarylethers (ID: 12) appeared in 47.2% and 34.0% of QT drugs, respectively, while their proportions in the no-DIQT-concern drugs were only 17.9% and 11.6%, respectively. Aryl halides (ID: 15) appeared in 37.5% and 13.7% of the QT-prolonging drugs and no-DIQT-concern drugs, respectively. In addition, Sp3-hybridized carbon atoms (2) (ID: 2) appeared in a higher proportion of QT-prolonging drugs, reaching 81.3%, while their proportion in the no-DIQT-concern drugs was 37.9%. Figure 1 illustrates the distribution of tertiary amines, alkylarylethers, and aryl halides among different therapeutic categories of QT-prolonging drugs. All three of these SAs were predominantly found in drugs classified under the therapeutic category of the nervous system (N), followed by those in the therapeutic categories of anti-infectives for systemic use (J) and antineoplastic and immunomodulating agents (L).

### 2.2. SAR Model Performance on DIQTA Dataset

**Figure 2:**
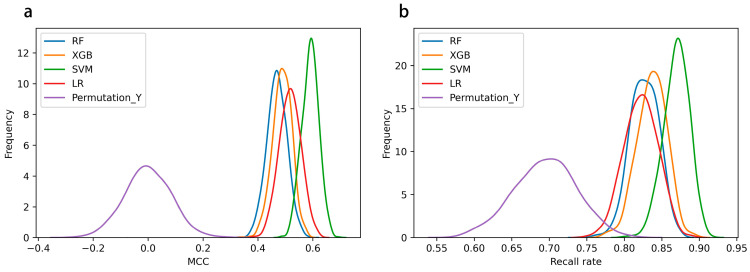
MCCs and recall rates achieved by SMV, LR, RF, and XGBoost with 1000 iterations of five-fold cross-validation and by SVM with permutation dataset. (**a**) The distribution of MCCs achieved by four models and SVM with permutation dataset. (**b**) The distribution of recall rates achieved by four models and SVM with permutation dataset.

Based on the DIQTA dataset, we used traditional machine learning methods, including logistic regression (LR), random forest (RF), support vector machine (SVM), and XGBoost, to develop the SAR model. The model was built using five-fold cross-validation, and the modeling process was repeated 1000 times to calculate the mean and variance of each prediction metric. Figure 2 shows the distribution of Matthews correlation coefficients (MCCs) and recall rates achieved by each algorithm. As shown in the figure, SVM achieved the highest MCC value and recall rate among all of the models. The detailed results are listed in Table 2, including accuracy, precision, recall score, MCC, f1 score, balanced accuracy score (BACC), the area under the ROC curve (AUC), average precision score (AP), sensitivity (SE), and specificity (SP). From the table, it can be seen that based on chemical structure information, each model achieved a predictive accuracy of more than 75% in predicting drugs with a potential risk of QT interval prolongation, indicating that each model can effectively predict the potential risk of the QT prolongation of drugs based on chemical structure information. Among them, SVM achieved the highest accuracy of 80.6%. Meanwhile, the recall rate for positive samples (drugs with risk of QT interval prolongation) was also the highest, reaching 87.0%. In addition, we also used SVM with 1000 iterations of five-fold cross-validation to build a model and predict the permutation data. As shown in Figure 2 and Table 2, for randomly labeled datasets, the prediction results of SVM were similar to the random results.

**Figure 3:**
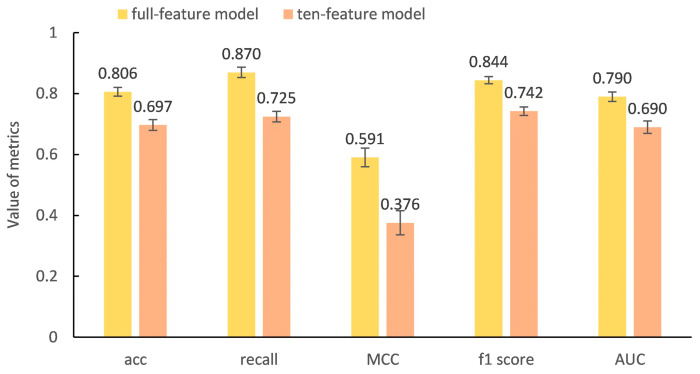
The predictive results achieved by SVM model with full features and ten features related to the 24 structural alerts.

Next, we used SVM and 24 SAs with more than 20% differences between QT-prolonging drugs and no-DIQT-concern drugs as features to identify drugs with the potential risk of inducing QT interval prolongation. The model was still constructed using 1000 iterations of five-fold cross-validation. We extracted the top 50 molecular descriptors ranked by their feature importance scores in descending order in SVM modeling (see Supplementary Table S1), and selected 10 of these molecular descriptors as features that were closely associated with the 24 SAs (see Table 3). Subsequently, the ten features were used to construct a SAR model to classify QT-prolonging drugs and no-DIQT-concern drugs. Figure 3 showed the prediction results using all features and only the ten features related to SAs. As can be seen from the figure, although the predictive accuracy, recall rate, and MCC of the model decreased after reducing the number of features, they were still 69.7%, 72.5%, and 0.376, respectively, indicating that using SAs as structural features can effectively identify drugs with a potential risk of QT interval prolongation.

### 2.3. SAR Model Performance on FAERS Dataset

We extracted 647 marketed drugs from the FAERS database that had at least one case report of inducing QT interval prolongation. We used SVM models established in the previous step to predict the potential risk of these drugs causing QT interval prolongation. Since the drugs with no DIQT concerns were not included in this dataset, we calculated the recall rate of the models. We sorted the 647 drugs by their reporting odds ratio (ROR) in descending order and took the top *N* drugs to construct a data subset. We used the models to predict the potential risk of QT interval prolongation for the drugs in the data subset, with *N* starting at 50 and increasing by 50 drugs each time until all drugs were included. This procedure allowed us to investigate the model performance in identifying drugs with different risks of QT interval prolongation.

**Figure 4:**
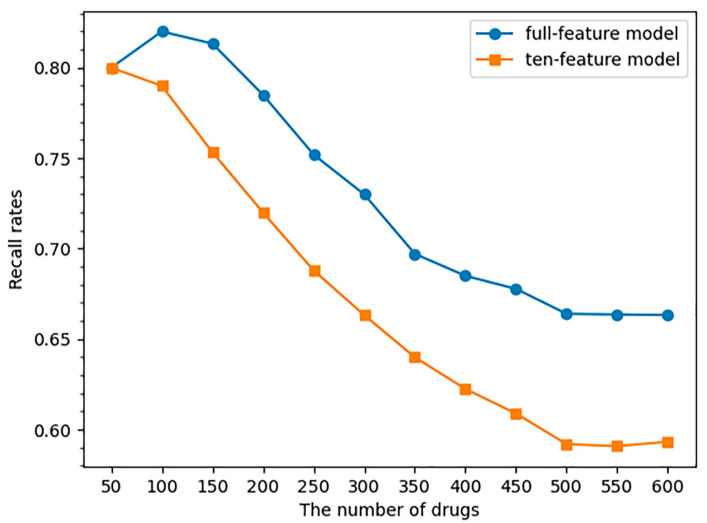
The trend of recall rate changed when the number of drugs increased in the dataset. The Y-axis indicates the recall rate, and the X-axis is the number of drugs used for the prediction.

Figure 4 showed the predictive performance of the models using all features and the models using ten SA-related features. Similarly to the results obtained via the DIQTA dataset, the predictive performance of the model with all features was higher than that of the ten-feature model. Both models achieved an 80.0% recall rate for identifying high-risk QT-prolonging drugs (the top 50 drugs with high ROR values). As more low-risk QT-prolonging drugs were included in the data subset, the recall rate of the full-feature model and the ten-feature model decreased from 80.0% to 66.3% and 59.3%, respectively, indicating that the models performed better in identifying high-risk QT-prolonging drugs compared to identifying low-risk QT-prolonging drugs. The top ten QT-prolonging drugs with the highest ROR values are listed in Table 4, of which eight drugs were correctly predicted as being positive. However, due to the limited number of features used, the ten-feature model could not effectively identify low-risk QT-prolonging drugs.

To further validate the predictive performance of the SVM models, we selected the top 95 QT drugs with the highest ROR values as positive samples from the FAERS database and 95 drugs that had not been reported to induce QT interval prolongation as negative samples from the DIQTA database. The therapeutic categories of the 95 no-DIQT-concern drugs were selected to be as consistent as possible with those of the 95 QT-prolonging drugs. We used this dataset as an independent test set to further validate the full-feature model and the ten-feature model. The results showed that the predictive accuracy of the full-feature model and the ten-feature model were 85.3% and 73.3%, and the recall rates were 81.1% and 77.9%, respectively, indicating that both models can effectively predict the potential risk of prolonging the QT interval for marketed drugs.

## 3. Discussion

Cardiotoxicity is a major concern in clinical medications, and drug-induced QT interval prolongation is a common occurrence in many therapeutic treatments, which may develop into severe arrhythmias, such as TdP. It is essential to establish a reliable strategy for assessing the potential risk of inducing QT interval prolongation of drugs. FDA-approved drug labeling information is determined after reviewing data from clinical trials and post-marketing surveillance, and can reflect the severity of the risk of QT interval prolongation induced by marketed drugs. In previous studies, we stratified QT-prolonging drugs and no-DIQT-concern drugs based on FDA-approved drug labeling information and constructed the DIQTA database. In this study, we compared the differences in chemical structures between QT-prolonging drugs and no-DIQT-concern drugs in the DIQTA database and identified three categories of SAs, namely amines, ethers, and aromatic compounds, related to the high risk of QT interval prolongation. The proportion of these SAs in the structure of QT-prolonging drugs is more than 20% higher than that in the no-DIQT-concern drugs, with the proportion of amines in QT-prolonging drugs being at least 30% higher than that in non-QT-prolonging drugs (Table 1). In the DIQTA database, more than 80% (79/96) of the most-DIQT-concern drugs (drugs that might cause fatal or life-threatening arrhythmia) contained these SAs, indicating that these three categories of SAs are closely related to the occurrence of drug-induced QT interval prolongation. In previous studies, it had been reported that the presence of tertiary amines, furan rings, and acetylene functional groups may inhibit the cytochrome P450 (CYP) 3A4 pathway, leading to blocked drug metabolism and accumulation in the body, ultimately causing cardiotoxicity [3,19,32,33,34]. For instance, terfenadine, a drug used to treat allergy symptoms, may cause serious side effects including delayed cardiac repolarization and ventricular tachycardia due to the accumulation of terfenadine in the body when CYP3A4 activity was inhibited [35].

The 24 SAs related to high-risk QT interval prolongation could be useful in gaining a better understanding of the mechanisms of drug-induced QT interval prolongation. The adverse outcome pathway (AOP) for DIQT provides a conceptual framework that connects the molecular initiating events (MIEs) to adverse outcomes (AOs) through a series of key events (KEs). The compounds that contain the three categories of substances of concern identified in our study could serve as chemical initiators, which trigger downstream KEs, such as ion channel inhibition, leading to QT prolongation. The identification of chemical initiators may guide the design of more effective in vitro and in vivo assays for predicting DIQT. Moreover, our findings could be utilized to develop risk assessment strategies for drug combinations that have the potential to cause QT interval prolongation.

In subsequent analysis, we used ten structural descriptors related to these 24 SAs as features to establish an SVM model and predict the potential risk of drugs inducing the prolongation of the QT interval. For the DIQTA dataset, the ten-feature model had a prediction accuracy and recall rate of 69.7% and 72.5%, respectively, which were lower than the prediction accuracy and recall rate (80.6% and 87.0%, respectively) obtained by the full-feature model. However, for the prediction of drugs with the potentially high risk of QT prolongation in the FAERS dataset (the top 50 drugs with high ROR values), both models achieved a recall rate of 80.0%, indicating that the ten-feature model has good performance in identifying drugs with a high risk of QT prolongation. Finally, for an independent test set containing 95 QT-prolonging drugs and 95 no-DIQT-concern drugs, the ten-feature model achieved a prediction accuracy of 73.7% and a recall rate of 77.9%. These results indicated that the 24 SAs revealed in this study might facilitate artificial-intelligence-based modeling for cardiotoxicity.

Additionally, there are some caveats that warrant further discussion. First, in the DIQTA database, QT-prolonging drugs and the drugs with no DIQT concerns were stratified based on FDA-approved drug labeling. If drug labeling from other countries, such as European Union (EU) countries, were used, there may be differences in the stratification of QT-prolonging drugs, which could ultimately lead to differences in SA identification. Secondly, our study instead revealed an association of a causality between the three categories of SAs and a high risk of drug-induced prolongation of the QT interval. The presence of these three categories of SAs in drug structures does not necessarily mean that the drugs will definitely cause QT interval prolongation or other cardiac toxicity. It only indicates that the drugs may have a higher likelihood of inducing QT interval prolongation and should be further evaluated for potential safety concerns. Finally, DIQT is a complex and multifactorial phenomenon. Other patient risk factors such as age, sex, and electrolyte imbalances can still cause QT interval prolongation during pharmaceutical treatment. Even though the chemical structure of a drug is one of the main factors that induces QT interval prolongation, it is still a challenge to establish a reliable machine learning model that predicts the risk of DIQT only based on chemical structures. It can be seen in Figure 4 that the recall rate for predicting low-risk QT-prolonging drugs achieved by the ten-feature model was only 59.3%. Therefore, it may be more effective to establish a model in future studies that combines multiple features, including clinical factors, to predict the risk of DIQT.

## 4. Materials and Methods

### 4.1. Study Design

**Figure 5:**
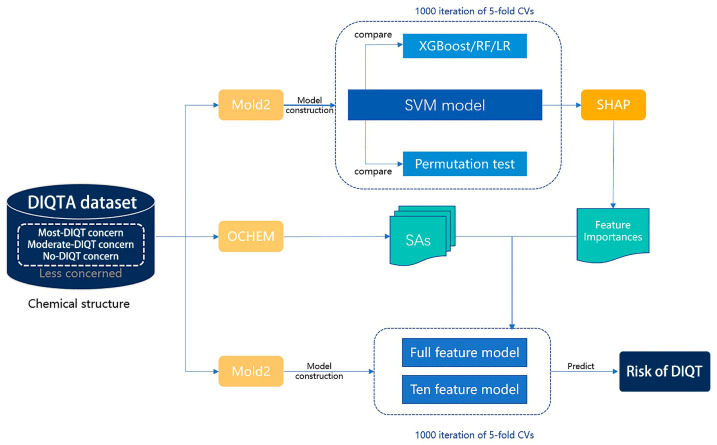
Workflow of our study.

In this study, we compared the differences in chemical structures between QT-prolonging drugs and drugs with no DIQT concerns, and identified 24 SAs that were closely associated with QT interval prolongation. Next, we utilized the Mold2 package to convert the chemical structures of the drugs into molecular descriptors, and established an SAR model based on the molecular descriptors related to the 24 SAs as features to predict the potential risk of prolonging the QT interval for drugs. Finally, the model was validated using the FAERS dataset. Figure 5 depicts the process in detail.

### 4.2. DIQTA Dataset

The Drug-Induced QT Prolongation Atlas (DIQTA, https://www.adratlas.com/DIQTA/, accessed on 22 February 2023) [31] stratified marketed drugs into different categories based on the severity of their effects on QT interval, including most-DIQT-concern drugs, moderate-concern drugs, ambiguous drugs, and no-DIQT-concern drugs. For structure comparison and model construction, we collected 144 drugs from the most-DIQT-concern and moderate-DIQT-concern categories as positive samples and 95 drugs from the no-DIQT-concern drugs as negative samples for this study.

### 4.3. FAERS Dataset

The FDA Adverse Event Reporting System (FAERS) primarily contains post-marketing surveillance data related to adverse events and medication errors associated with FDA-regulated drugs [36]. We collected all reported cases from 2004 to 2021 for this study, resulting in a total of 38,405,679 drug-event pairs. Please refer to our previous studies for detailed information on the generation procedures [31,37]. We extracted 903 drugs reported to have induced QT interval prolongation and identified 647 drugs with available chemical structures for subsequent analysis.

To assess the severity of drug-induced QT interval prolongation for each drug, we calculated the reporting odds ratio (ROR) using the following formula:

(1) $$\mathrm{ROR}=\frac{\left(\frac{a}{c}\right)}{\left(\frac{b}{d}\right)}$$

where a, b, c, and d are the numbers of cases defined in the Table 5.

Based on their ROR values, the 647 drugs were ranked in ascending order and used for SAR model validation.

### 4.4. Identification of Structural Alerts

After uploading SDF files for both QT-prolonging drugs and no-DIQT-concern drugs to the ToxAlert platform in the Online Chemical Database (OCHEM, https://ochem.eu/home/show.do, accessed on 22 February 2023) [38,39], we separately obtained structural alerts (SAs) present in each category of drugs. In total, we identified 602 SAs from the structures of QT-prolonging drugs, along with a list of drugs that contained each SA. We also obtained 669 SAs from the no-DIQT-concern drugs and their corresponding drug lists. By dividing the number of drugs in a specific SA’s list by the total number of drugs in the category (QT or no-DIQT-concern drugs), we were able to calculate the proportion of each SA in each of the two categories. We retained an SA if its proportion in QT-prolonging drugs was 20% higher than its proportion in non-QT-prolonging drugs. As a result, we kept 24 SAs for further analysis.

### 4.5. Calculation and Selection of Molecular Descriptors

The calculation of molecular descriptors was conducted using the Mold2 package (https://www.fda.gov/science-research/bioinformatics-tools/mold2, accessed on 22 February 2023), which was developed by Dr. Tong at the National Center for Toxicological Research (NCTR), US FDA [40,41,42,43]. The package provided a collection of 777 1D and 2D molecular descriptors, and we utilized it to calculate the molecular descriptors for each drug in the DIQTA and FAERS datasets. We applied a variance threshold of 0.001 to remove descriptors with low variability among the drugs, resulting in a final set of 603 descriptors for SAR modeling. The input data were uploaded to GitHub (https://github.com/LiSH7450/DIQT_model, accessed on 20 March 2023).

### 4.6. SAR Model Construction

We used the support vector machine (SVM) algorithm [44] to predict the risk of drug-induced QT prolongation based on the chemical structure of the drug. Based on the DIQTA dataset, the SVM model with an RBF kernel was constructed by using 603 molecular descriptors as features and five-fold cross-validation. The regularization parameter (c) and the kernel width (γ) were optimized using a grid search method. Drugs with predicted probabilities greater than 0.5 were considered to have a higher risk of causing QT interval prolongation. To assess the robustness of the model, we conducted 1000 iterations of the modeling process. To evaluate the performance of the model, we compared the performance with other machine learning models, namely random forest (RF) [45], logistic regression (LR) [46], and extreme gradient boosting (XGBoost) [47]. The parameters of these models were also optimized using a grid search method, and all models were constructed using 1000 iterations of five-fold cross-validation. Additionally, we created a permutation dataset as a negative control for SVM model construction by randomly permuting the labels of samples.

### 4.7. Feature Selection

We evaluated the importance of each feature in an SVM model construction procedure using the Shapley additive explanations (SHAP) algorithm, which was introduced by Lundberg and Lee [48]. This algorithm was proposed to explain a model’s output by assigning a score to each input feature, indicating how much that feature contributes to the model’s prediction for a particular instance. We extracted the top 50 features that contributed the most to the prediction results, and 10 out of the 50 features were found to be closely associated with the 24 SAs. These ten features were used to construct the SVM model. A ten-feature model was then constructed using the ten identified molecular descriptors as features and 1000 iterations of five-fold cross-validation.

### 4.8. Evaluation of Model Performance

To evaluate the performance of the model, we used nine performance metrics in this study, including accuracy, precision, recall score, Matthews correlation coefficient (MCC), f1 score, balanced accuracy score (BACC), the area under the ROC curve (AUC), average precision score (AP), sensitivity (SE), and specificity (SP).

(2) $$\mathrm{accuracy}=\frac{\mathrm{TP}+\mathrm{TN}}{\mathrm{TP}+\mathrm{TN}+\mathrm{FP}+\mathrm{FN}}$$

(3) $$\mathrm{recallrate}=\frac{\mathrm{TP}}{\mathrm{TP}+\mathrm{FN}}$$

(4) $$\mathrm{precision}=\frac{\mathrm{TP}}{\mathrm{TP}+\mathrm{FP}}$$

(5) $$\mathrm{MCC}=\frac{\mathrm{TP}\times \mathrm{TN}-\mathrm{FP}\times \mathrm{FN}}{\sqrt{\left(\mathrm{TP}+\mathrm{FP}\right)\left(\mathrm{TP}+\mathrm{FN}\right)\left(\mathrm{TN}+\mathrm{FP}\right)\left(\mathrm{TN}+\mathrm{FN}\right)}}$$

(6) $$F1\mathrm{score}=\frac{2\times \mathrm{precision}\times \mathrm{recall}\;\mathrm{rate}}{\mathrm{precision}+\mathrm{recall}\;\mathrm{rate}}$$

(7) $$\mathrm{BACC}=\frac{\left(\mathrm{TPR}+\mathrm{TNR}\right)}{2}$$

(8) $$\mathrm{AUC}=\int_{x=0}^{1}\mathrm{TPR}\left({\mathrm{FPR}}^{-1}\left(x\right)\right)\mathrm{dx}$$

(9) $$\mathrm{AP}=\int_{-\infty }^{+\infty }\mathrm{precision}\left(x\right)\mathrm{dP}\left[Y\leq x\right]$$

(10) $$\mathrm{SE}=\frac{\mathrm{TP}}{\mathrm{TP}+\mathrm{FN}}$$

(11) $$\mathrm{SP}=\frac{\mathrm{TN}}{\mathrm{TN}+\mathrm{FP}}$$

TP, TN, FP, and FN stand for true positive, true negative, false positive, and false negative, respectively. To evaluate the performance of the model, we conducted 5-fold cross-validation and averaged the results over 1000 iterations.

## 5. Conclusions

In this study, we discovered a total of 24 SAs that are associated with high-risk QT-prolonging drugs. The compounds containing these SAs can be used as molecular initiating events to enhance the adverse outcome pathway related to QT interval prolongation. By using these SAs as features, we can establish an SVM to effectively predict high-risk QT-prolonging drugs. Our findings are expected to significantly improve research on cardiac adverse drug reactions in drug development and reduce drug attrition due to drug-induced prolongation of the QT interval.

## Figures and Tables

**Table 1:** Twenty-four chemical fragments (SAs) with large differences in chemical structures between QT-prolonging drugs and no-DIQT-concern drugs.

ID	Class	Name	SA	Number of QT-Prolonging Drugs	Proportion of QT-Prolonging Drugs	Number of Non-QT-Prolonging Drugs	Proportion of Non-QT-Prolonging Drugs	Difference
1	amines	tertiary amines	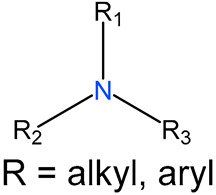	88	0.6111	12	0.1263	0.4848
2		sp3-hybridized carbon atoms (2)	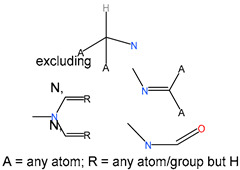	117	0.8125	36	0.3789	0.4336
3		tertiary aliphatic amines	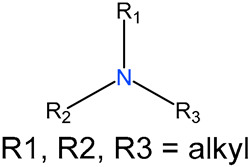	76	0.5278	9	0.0947	0.4330
4		16-tertiary amine	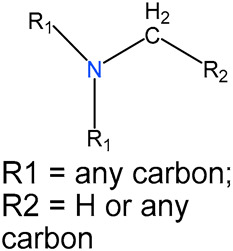	91	0.6319	19	0.2	0.4319
5		amines	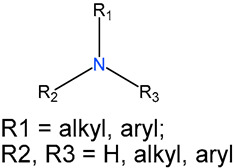	119	0.8264	38	0.4	0.4264
6		B3-tertiary amine	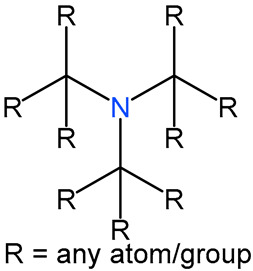	76	0.5278	10	0.1053	0.4225
7		nitrogen atoms (1)	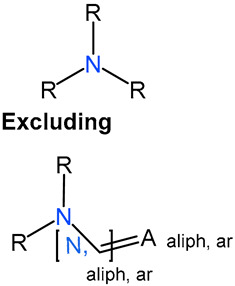	78	0.5417	12	0.1263	0.4154
8		36-CH2N	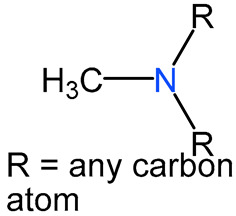	65	0.4514	14	0.1474	0.3040
9	ethers	ethers	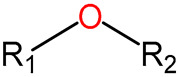	68	0.4722	17	0.1789	0.2933
10		sp3-hybridized carbon atoms (6)	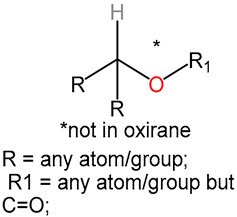	86	0.5972	32	0.3368	0.2604
11		13-ether	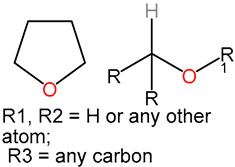	65	0.4514	20	0.2105	0.2409
12		alkylarylethers	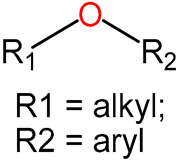	49	0.3403	11	0.1158	0.2245
13	aromatic compounds	arenes	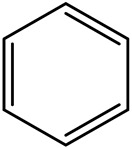	127	0.8819	56	0.5895	0.2925
14		11-AC(3-Aromatic carbon)	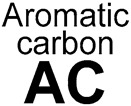	119	0.8264	55	0.5789	0.2474
15		aryl halide	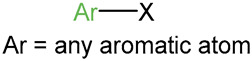	54	0.375	13	0.1368	0.2382
16		4-aromatic carbon-alkane	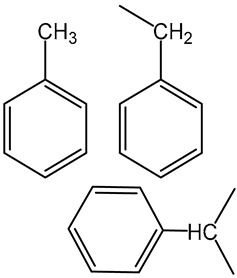	81	0.5625	31	0.3263	0.2362
17		aromatichalogen	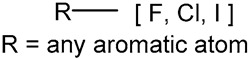	53	0.3681	13	0.1368	0.2312
18		10-ACH (3-aromatic carbon)	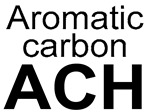	129	0.8958	65	0.6842	0.2116
19	others	base	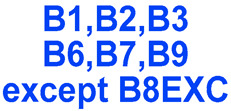	126	0.875	49	0.5158	0.3592
20		six-membered heterocycles with one heteroatom (LS)	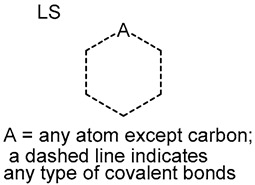	69	0.4792	21	0.2211	0.2581
21		2-CH_2_ (1-Alkane group)	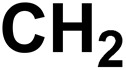	130	0.9028	63	0.6632	0.2396
22		halogen derivatives	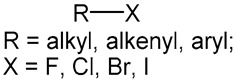	66	0.4583	22	0.2316	0.2268
23		halogens	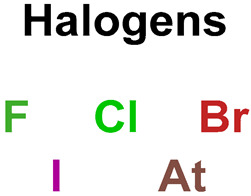	66	0.4583	22	0.2316	0.2268
24		NUC	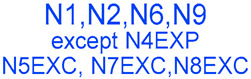	91	0.6319	40	0.4211	0.2109

**Table 2:** Prediction results of RF, XGBoost, SVM, LR, and Permutation_Y.

	XGBoost	RF	LR	SVM	Permutation_Y
Accuracy	0.758 ± 0.016	0.749 ± 0.017	0.770 ± 0.019	0.806 ± 0.014	0.540 ± 0.037
Recall score	0.837 ± 0.020	0.827 ± 0.019	0.822 ± 0.022	0.870 ± 0.017	0.695 ± 0.041
Precision score	0.779 ± 0.014	0.773 ± 0.015	0.801 ± 0.017	0.820 ± 0.014	0.602 ± 0.027
MCC	0.488 ± 0.034	0.469 ± 0.036	0.517 ± 0.039	0.591 ± 0.031	−0.001 ± 0.084
BACC	0.738 ± 0.017	0.729 ± 0.018	0.756 ± 0.019	0.790 ± 0.015	0.500 ± 0.039
F1 score	0.807 ± 0.013	0.799 ± 0.014	0.811 ± 0.016	0.844 ± 0.012	0.645 ± 0.030
AUC	0.738 ± 0.017	0.729 ± 0.018	0.756 ± 0.019	0.790 ± 0.015	0.500 ± 0.039
AP	0.750 ± 0.013	0.744 ± 0.014	0.766 ± 0.016	0.791 ± 0.013	0.603 ± 0.019
SE	0.837 ± 0.020	0.827 ± 0.019	0.822 ± 0.022	0.870 ± 0.017	0.695 ± 0.041
SP	0.640 ± 0.028	0.632 ± 0.032	0.691 ± 0.032	0.710 ± 0.026	0.305 ± 0.061

**Table 3:** Ten of these molecular descriptors as features that were closely associated with the 24 SAs.

Descriptor	Description
D718	number of CH_3_X groups
D756	number of Al-O-Ar or Ar-O-Ar or R-O-C=X groups
D661	number of quaternary ammonium (aliphatic) groups
D759	number of tertiary aliphatic amine groups
D627	number of tertiary amides (aliphatic) groups
D130	number of halogen atoms in each molecule
D647	number of primary amines (aliphatic) groups *
D626	number of secondary amides (aromatic) groups
D757	number of Al-NH_2_ groups
D598	number of total tertiary C-sp^3^

* Aliphatic primary amines have a carbon with sp3 hybridization and two hydrogen atoms connected to the nitrogen atom.

**Table 4:** The top ten QT-prolonging drugs with the highest ROR values.

Drug Name	Odds Ratio	ATC Code
Doxapram	398.298	R07
Cisapride *	272.797	A03
Ibutilide *	223.102	C01
Tropisetron *	113.799	A04
Trimebutine *	113.799	A03
Alfacalcidol *	106.213	M05, A11
Bedaquiline *	97.265	J04
Ethionamide	79.660	J04
Bepridil *	78.360	C08
Procainamide *	59.457	C01

* Drugs that were predicted as positives by our model.

**Table 5:** Contingency table for calculating the ROR.

	Cases with Current ADR	Cases without Current ADR
Cases with current drugs	a	b
Cases without current drugs	c	d

## Data Availability

The data presented in this study are openly available in DIQTA at https://doi.org/10.1016/j.drudis.2021.10.009, accessed on 22 February 2023, reference number [31].

## Supplementary Materials

The following supporting information can be downloaded at: https://www.mdpi.com/article/10.3390/ijms24076771/s1.
